# Genetic Alterations and Deregulation of Hippo Pathway as a Pathogenetic Mechanism in Bone and Soft Tissue Sarcoma

**DOI:** 10.3390/cancers14246211

**Published:** 2022-12-15

**Authors:** Carmen Salguero-Aranda, Joaquín Olmedo-Pelayo, Enrique de Álava, Ana Teresa Amaral, Juan Díaz-Martín

**Affiliations:** 1 Institute of Biomedicine of Sevilla (IBiS), Virgen del Rocio University Hospital/CSIC/University of Sevilla, 41013 Seville, Spain; 2 Centro de Investigación Biomédica en Red de Cáncer, Instituto de Salud Carlos III (CB16/12/00361, CIBERONC-ISCIII), 28029 Madrid, Spain; 3 Department of Normal and Pathological Cytology and Histology, School of Medicine, University of Seville, 41009 Seville, Spain

**Keywords:** Hippo pathway, YAP, TAZ, sarcoma, gene fusion

## Abstract

**Simple Summary:**

Cancer is a genetic disease that is caused by changes in genes controlling cell growth, migration, and differentiation. Usually, cancer cells hijack processes used by healthy cells during organism development. The Hippo pathway is a developmental signaling system with a critical role in tissue and organ size regulation, which is frequently deregulated in cancer. Indeed, the contribution of Hippo dysfunction to cancer development has been extensively reported in carcinomas, but it is increasingly recognized in sarcomas. Sarcomas are rare cancers that develop in the bones and soft tissues, encompassing a large variety of different subtypes. Here we review the relevance of the Hippo pathway in specific sarcoma subtypes, with a focus on both the genetic alterations in Hippo pathway genes as well as other molecular mechanisms involved in its deregulation.

**Abstract:**

The Hippo pathway is an evolutionarily conserved modulator of developmental biology with a key role in tissue and organ size regulation under homeostatic conditions. Like other signaling pathways with a significant role in embryonic development, the deregulation of Hippo signaling contributes to oncogenesis. Central to the Hippo pathway is a conserved cascade of adaptor proteins and inhibitory kinases that converge and regulate the activity of the oncoproteins YAP and TAZ, the final transducers of the pathway. Elevated levels and aberrant activation of YAP and TAZ have been described in many cancers. Though most of the studies describe their pervasive activation in epithelial neoplasms, there is increasing evidence pointing out its relevance in mesenchymal malignancies as well. Interestingly, somatic or germline mutations in genes of the Hippo pathway are scarce compared to other signaling pathways that are frequently disrupted in cancer. However, in the case of sarcomas, several examples of genetic alteration of Hippo members, including gene fusions, have been described during the last few years. Here, we review the current knowledge of Hippo pathway implication in sarcoma, describing mechanistic hints recently reported in specific histological entities and how these alterations represent an opportunity for targeted therapy in this heterogeneous group of neoplasm.

## 1. Introduction

The Hippo pathway is an evolutionary and functionally conserved pathway that controls developmental processes, differentiation, and regeneration by regulating organ size and tissue homeostasis [1,2]. This pathway was initially discovered in *Drosophila melanogaster* due to tumor suppressor screens and was later revealed to be conserved in mammals. These studies identified *Warts* (*Wts*, *LATS1/2* in humans) [3,4] and *Hippo* (*Hpo*, or *STK4/3* encoding MST1/2 in humans) [5,6] genes, which encode the kinases that constitute the principal phosphorylation cascade to the signaling pathway. Likewise, in flies, Hippo mutants display phenotypes of extremely sized organs and apparently resemble a hippopotamus, naming this signaling pathway as it is currently known—the Hippo pathway [5]. 

In recent years, aberrations on the Hippo pathway have been increasingly associated with cancer development. Thus, many studies have experimentally established its tumor suppressor function. For example, *Mst1/2* loss leads to uncontrolled cell proliferation and differentiation in a mouse liver [7], and *Yap1/Taz* overexpression, the transcriptional coactivators of the pathway, triggers tissue overgrowth and cancer [8,9]. Therefore, dysregulation of the hippo pathway has been reported in various cancer types, including sarcomas [7,10,11,12,13,14,15,16], and correlated with poor prognosis [17]. This review will focus on the genomic alterations disturbing the Hippo pathway and how these aberrations might be potential therapeutic targets in bone and soft tissue sarcomas.

## 2. The Hippo Signaling Pathway: Critical Components in Mammals and Basic Biology

The primary function of the Hippo pathway is to inhibit proliferation and promote apoptosis, thereby controlling organ growth [18]. This role is arbitrated by a cascade of kinases that transmit, from the plasma to the nucleus, various upstream mechanical, architectural, and metabolic signals. 

The Hippo regulating plasma membrane proteins principally include E-cadherin (CHD1) [19], protocadherin FAT4 [20], wingless-related integration (WNT) [21,22,23], the Crumbs polarity complex [24], LIM domain-containing protein Ajuba (AJUBA) [25], the hyaluronic acid receptor CD44 [26], and G-protein coupled receptors (GPCR) [27]. These proteins control the members of the upstream intracellular pathway, which include neurofibromatosis type 2 (NF2), also known as merlin [28], kidney and brain protein (KIBRA or WWC1) [29], Ras-association domain family members (RASSF1–10) [30], TAO kinases (1–3) [31] and angiomotin (AMOT) [32]. All these upstream regulators play a vital role in initiating the cascade of phosphorylation of the core Hippo pathway members.

When the Hippo pathway is activated, the STE20-like kinase 1/2 (MST1/2) is phosphorylated on threonine 183/180, mainly by TAO kinases [33], although it has been described that the activation can be achieved by MST1/2 autophosphorylation itself [34]. Active MST1/2 then phosphorylates the large tumor suppressor kinase 1/2 (LATS1/2) protein [35], but LATS1/2 can also be directly activated by the upstream regulators NF2, AJUBA, and TAO kinases [28,36,37]. MST1/2 also phosphorylates the Salvador family WW domain-containing protein 1 (SAV1) and MOB kinase activator 1A and 1B (MOB1A/B), which are scaffold proteins that coordinate the phosphorylation of MST1/2 and LATS1/2 protein kinases [38,39]. In turn, active LATS1/2 phosphorylates the paralogous transcriptional cofactors Yes-associated protein 1 (YAP) (gene symbol, *YAP1*) and PDZ-binding motif (TAZ) (gene symbol, *WWTR1*) on the serine S127 and S89, respectively, which results in their inactivation through translocation from the nucleus to the cytosol, binding with 14-3-3 protein and proteasomal degradation [40,41]. Thus, the cofactors TAZ and YAP are negatively regulated by the Hippo pathway.

When the Hippo signaling pathway is inactivated, non-phosphorylated YAP or TAZ are stabilized and translocated into the nucleus. Because of the lack of DNA-binding domains of YAP/TAZ, they require to cooperate with DNA-binding transcription factors to induce the expression of genes involved in cell proliferation, migration, survival, tissue growth, and inhibition of apoptosis [42]. YAP and TAZ interact preferentially with transcriptional enhanced associate domain (TEAD) proteins (TEAD1–4) [43,44] but also with other transcription factors such as SMAD family members [45,46], Erb-B2 receptor tyrosine kinase 4 (ERBB4) [47], T-box transcription factor 5 (TBX5) [48,49], RUNX family transcription factor 1, 2 and 3 (RUNX1/2/3) [50,51], early-growth response 1 (EGR1) [52], hypoxia-inducible factor 1 alpha (HIF1Aα) [53], core-binding factor subunit beta (CBFB) (also called PEBP2) [54] and tumor protein p73 [55]. Depending on the binding of YAP to one of these DNA-binding transcription factors and, subsequently, associated promoters, diverse target genes are activated. For example, target genes of the YAP/TAZ-TEAD complex include *CYR61*, *CTGF*, *AREG*, or *MYC*; YAP-TBX5 complex induces the expression of transcriptional targets such as *BCL2L1* and *BIRC5*; and YAP-ERBB4 regulates the expression of *CTGF*, *CYR61*, and *ANKRD1* [42], involved in cell proliferation, growth, migration, and survival. 

In addition to the central inhibitory kinase core, the regulation of YAP and TAZ activity is also controlled by multiple Hippo-independent mechanisms. There is extensive crosstalk with other pathways that influence YAP/TAZ activity beyond the canonical Hippo pathway, such as WNT signaling, TGFβ signaling, GPCR, Rho GTPases or tyrosine kinases-PI3K-AKT signaling [56,57]. Of note, the prominent role of YAP and TAZ integrating morphogenic signals in mechanotransduction processes is modulated both by Hippo-dependent and independent mechanisms. The organization of the actin cytoskeleton seems to be the main input of mechanical cues involving Rho-family GTPases and ROCK (Rho-associated protein kinase) proteins that control F-actine polymerization and ultimately affect YAP/TAZ activity in a LATS-dependent or independent manner [57,58]. Moreover, cell-substratum interaction mediated by integrins promotes the activation of YAP/TAZ by SRC kinase. Indeed, SRC and other SRC family kinases can activate YAP/TAZ through multiple mechanisms, including direct phosphorylation conferring protein stability, enhancing transcriptional activity, and/or interaction with other transcription factors. SRC-mediated activation of YAP/TAZ can also occur through repression of LATS or Hippo pathway-independent mechanisms [59,60]. 

## 3. Deregulation of the Hippo Signaling Pathway in Bone and Soft Tissue Sarcoma

Given the critical role of the Hippo pathway in regulating these multiple cellular processes, it is not surprising that aberrant activation of YAP/TAZ leads to uncontrolled cell proliferation and malignant transformation. Indeed, cancer cells commonly hijack the Hippo pathway to acquire malignant properties. 

There is extensive evidence that increased expression of YAP/TAZ associates with tumor onset and progression in a large variety of cancers [17]. Actually, the Cancer Genome Atlas (TCGA) project that performed multi-omics profiling in a pan-cancer cohort of 9125 patients across 33 cancer types and characterization of 19 Hippo core genes indicated widespread deregulation of the Hippo pathway members in human cancers. Their main finding is that Hippo signaling is especially relevant in the pathogenesis of carcinomas with squamous cell differentiation. This was mainly attributed to the elevated proportion of cases with *YAP1/WWTR1* genomic amplification and high expression heterogeneity of YAP/TAZ target gene signature, which correlated with decreased overall survival of patients with squamous cell cancers. With regard to sarcomas, attending to this report, they seem to be among the malignancies with less genomic alterations in Hippo-related genes and exhibited a poor correlation between YAP/TAZ target gene signature and overall survival. The somatic copy number alteration study showed a significant deletion peak in 17p in sarcomas, where *TAOK1* resides [16]. However, it is important to bear in mind that the data analyzed corresponded to a small subset of sarcoma subtypes (leiomyosarcomas, dedifferentiated liposarcomas, and myxofibrosarcomas/undifferentiated pleomorphic sarcomas), which does not represent the enormous diversity of different entities. Besides, pooled analysis of different sarcoma entities may hinder specific features. Therefore, the functional relevance of Hippo signaling in different types of sarcomas should be evaluated in specific entities.

Sarcomas are a highly heterogeneous and complex group of mesenchymal malignancies, both in terms of morphology and pathobiology, that represent <1% of all malignant neoplasms in adults [61].The WHO classification of bone and soft tissue sarcoma listed approximately 100 different sarcomas and mesenchymal tumors of intermediate malignancy. From the genomic point of view, sarcomas can be broadly classified into two groups. Around 1/3 are translocation-associated sarcomas (t-sarcomas), mainly arising in children and young adults, and 2/3 are non-t sarcomas that display complex karyotypes with no specific genomic patterns. In the case of t-sarcomas, the translocation generates a specific fusion gene, which is the driver oncogene of the disease and is an important hallmark to differentiate between different neoplasms among the large variety of entities. In fact, t-sarcomas show an overall low mutational burden apart from gene fusion. Interestingly, several t-sarcomas exhibit recurrent translocations involving Hippo-related genes. Particularly, the genes *YAP1* and *WWTR1* are identified to be rearranged in certain subtypes of sarcomas and in other unrelated tumor types, such as supratentorial ependymoma (*YAP1::MAMLD1*, *YAP1::FAM118B*), cervical squamous cell carcinoma and endocervical adenocarcinoma (*YAP1::SS18*), poroma/porocarcinoma (*YAP1::MAML2*, *YAP1::NUTM1*), or NF2-wild type meningioma (*YAP1::MAML2*, *YAP1::FAM118B*, *YAP1::PYGO1*, *YAP1::LMO1*) [62]. Moreover, Hippo pathway deregulation mediates the oncogenic properties of other recurrent sarcoma gene fusions. Intriguingly, most of the reports describing the functional relevance of the Hippo pathway in sarcomas deal with t-sarcomas, despite the fact that they represent only 1/3 of the mesenchymal malignancies.

Several studies have demonstrated that the Hippo pathway is deregulated in sarcomas. For example, fusion genes involving *WWTR1* and *YAP1* are found in nearly all cases of epithelioid haemangioendothelioma [63,64]; *YAP1* copy number gain has been described in embryonal rhabdomyosarcoma [65] and frequent hypermethylation of *MST1*, *MST2* and *RASSF1A* has been shown in several subtypes of soft tissue sarcoma [66]. Furthermore, a study encompassing an immunohistochemistry (IHQ) assessment of TAZ and YAP in 159 sarcomas representing the most prevalent types showed that 50% and 66% of samples exhibit activation (or nuclear location) of YAP and TAZ, respectively [67]. A later study analyzed the expression levels of YAP and TAZ by IHQ in a cohort of 486 sarcoma tissues. Nuclear YAP and TAZ expression levels were detected in 53% and 33% to be moderate to intense, respectively [68]. Additionally, deregulation of the hippo pathway has been related to poor prognosis in several subtypes of sarcomas [67,69,70,71]. These pieces of evidence suggest that the Hippo pathway plays a crucial role in sarcoma tumorigenesis, progression, and outcome. 

**Figure 1:**
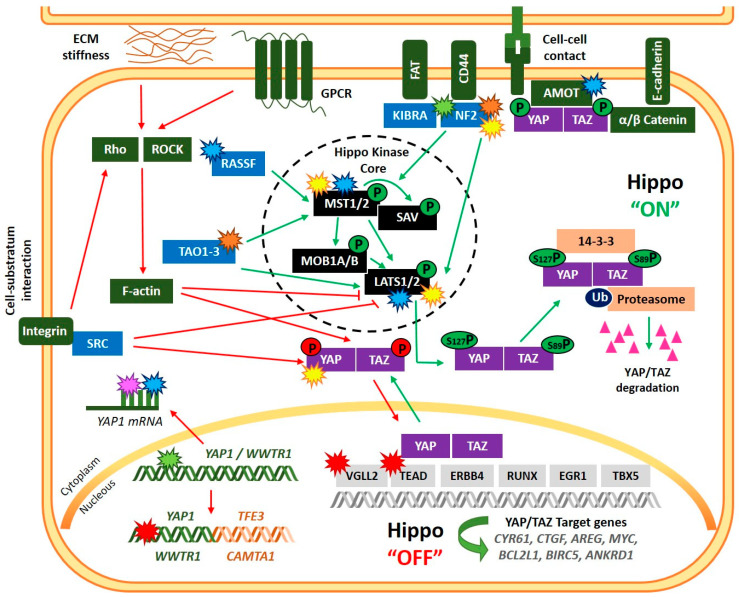
Regulation of the Hippo signaling pathway and main alterations of Hippo-pathway members reported in sarcomas. Green arrow lines and phosphates indicate induction of Hippo “ON” status, while red arrow lines and phosphates indicate “OFF” status. Alterations of the Hippo-pathway members are displayed with start symbols with colors denoting: transcriptional (green); post-transcriptional (pink); post-translational (yellow); epigenetic (blue), mutation/copy number alteration (orange) and chromosomal rearrangement (red) aberrations. An example of chromosomal rearrangement involving *YAP1* and *WWTR1* is showed in the figure (*YAP1::TFE3* and *WWTR1::CAMTA1*).

In this section, we will discuss alterations that affect Hippo pathway members in specific subtypes of sarcomas (Figure 1 and Table 1).

### 3.1. Osteosarcomas

Osteosarcoma (OS) is the most common primary malignancy of bone and one of the most common primary malignant tumors in children and adolescents. OS can occur in any bone, with 75% of all cases occurring in the distal femur and proximal tibia [127,128]. OS is characterized by heterogeneous genetic complexity, including complex genomic rearrangements as well as copy number alterations [129,130]. In addition, aberrations in the Hippo pathway have been extensively reported, and the deregulation of several members of this signaling pathway is described as tumorigenic factors in OS.

#### 3.1.1. YAP 

In 2013, Zhang et al. reported nuclear localization of YAP in OS patient tumor biopsies and that *YAP1* knockdown inhibited the proliferation and invasion of OS cells by downregulation of the RUNX2 pathway [131]. The association between YAP nuclear localization and a poor prognosis in OS was reported by Bouvier et al., who suggested that the Hippo pathway could represent a therapeutic target in conventional OS [70]. Additionally, the transcription factor TEAD1 has been reported to be involved in YAP-driven OS development. Genetic silencing of *TEAD1* suppresses several malignant phenotypes of OS cells, including cell proliferation, resistance to apoptosis, and invasiveness [132]. Interestingly, it has been shown that YAP and pSmad2 (a marker of active TGFβ signaling) have potential prognostic value in canine appendicular OS [133]. 

*YAP1* can be upregulated by Hedgehog (Hh) pathway activation. Chan L.H. et al. have reported that *YAP1* was overexpressed in both human and mouse tumor tissues and that *YAP1* expression was reduced by targeting the Hh signaling pathway. They also showed that the upregulation of the Hh signaling significantly prompted osteoblastic OS cells in mature osteoblasts. In addition, they described the aberrant expression of the long noncoding RNA (lncRNA), *H19*, and proved that its regulation was Hh signaling and YAP expression-dependent [72]. 

YAP can also be upregulated by the human HLA-F adjacent transcript 10 (FAT10) protein, a member of the ubiquitin-like protein family. It has been reported that FAT10 plays an essential role in developing malignant tumors and stabilizes YAP expression by modifying its ubiquitination and degradation. Moreover, this study revealed that FAT10 is overexpressed in OS, and in vivo and in vitro assays proved that FAT10 silencing inhibited OS proliferation [79]. 

A functional connection between Rho-associated coiled-coil containing protein kinase 2 (ROCK2) and YAP in regulating OS cell migration and metastasis formation has been described by Zucchini et al. They reported that ROCK2 silencing induced a reduction in the nuclear expression and transcriptional activity of YAP and significantly reduced tumor growth, and eradicated the metastatic potential of OS cell lines [80]. In this context, ROCK2 has been reported to be significantly upregulated in OS tissues compared with adjacent normal tissues. The expression level is related to tumor size and patient prognosis [81,82].

HuR, an RNA-binding protein, can also control *YAP1* expression. Thus, Li Z. et al. showed that the expression of HuR is meaningfully increased in OS tissues and positively correlates with OS progression. Moreover, the knockdown of *HuR* suppressed OS cell migration and invasion, the epithelial-mesenchymal transition (EMT) process, and the stemness of OS cells. Mechanistically, it was proved that HuR directly binds to *YAP1* mRNA, stabilizing and increasing its transcriptional activity. Significantly, *HuR* and *YAP1* expression was positively correlated in OS tissues [74]. A similar study by Xu, W., et al. revealed that the expression of the lncRNA, *B4GALT1-AS1*, was considerably increased in OS tissues. *B4GALT1-AS1* was found to recruit HuR to enhance *YAP1* mRNA stability and its transcriptional activity. *B4GALT1-AS1* knockdown repressed proliferation, migration, and stemness of OS cells. Importantly, in vitro and in vivo assays of *YAP1* overexpression rescued the inhibition of *B4GALT1-AS1* knockdown on OS cell progression [75]. 

Liu G. et al. have observed significant upregulation of *circFAT1*, a circular RNA originating from exon two of the *FAT1* gene, in human OS tissues and cell lines. In this study, the in vitro inhibition of *circFAT1* efficiently prevented the migration, invasion, and tumorigenesis of OS cells and repressed in vivo OS growth. Mechanistic studies showed that *circFAT1* could sponge *microRNA-375* (*miR-375*), which was found to be downregulated in OS tissues and cell lines. Furthermore, they described that *YAP1* 3′-UTR mRNA is directly targeted by *miR-375*, revealing other potential regulatory properties of the circularized protein-coding exons or “sponging miRNAs” and providing a new therapeutic target for the OS treatment [76]. 

Luo Y. et al. described the upregulated expression of *miR-624-5p* in OS cells and tissues. A higher malignant phenotype of OS was observed when overexpressing *miR-624-5p* in in vitro and in vivo assays. In addition, they revealed that the expression of the protein tyrosine phosphatase receptor type B (PTPRB) was negatively correlated and identified the Hippo signaling pathway to be involved in the *miR-624-5p*/PTPRB axis, although precise mechanisms demand further research [134]. 

An opposite role to that described for *miR-624-5p* has been reported for *miR-625*. Luo Z. et al. revealed that *miR-625* was markedly downregulated in OS tissues and cell lines. Mechanistically, they showed that *miR-625* mimic attenuated the cell proliferation and invasion of OS cells by directly binding to *YAP1* 3′-UTR mRNA and suppressing *YAP1* expression. Furthermore, *YAP1* upregulation rescued the inhibitory properties of *miR-625* on OS cell proliferation and invasion [77].

Cheng L. et al. have shown that Gankyrin, a regulatory subunit of the proteasome complex, is upregulated in OS and predicts disease progression and poor prognosis. Mechanistic studies revealed that gankyrin avoids *YAP1* downregulation mediated by *miR-200a* through P53 and origins a positive feedback loop to regulate YAP signaling in OS cells. Furthermore, in vitro and in vivo studies showed that gankyrin interacts with YAP to induce OS tumorigenesis [78].

#### 3.1.2. NF2 

NF2 has also been described as playing a role in OS development. In human, germline or somatic mutations in one allele of *NF2* results in the disease neurofibromatosis type 2, which is associated with schwannomas, meningiomas, and ependymomas. Nevertheless, heterozygous *Nf2* mutant mice develop mainly osteomas and OS [83,84]. 

NF2 activity depends on specific interaction with the cytoplasmic tail of CD44, a transmembrane hyaluronate receptor that functions as an upstream regulator sensing the extracellular environment to modulate ERK, AKT, and Hippo pathways [85,86]. A study carried out by Gvozdenovic A. et al. revealed that CD44 silencing in OS cells reduces the number of proliferative cells and decreases the content of NF2 protein. However, in vivo studies showed that OS cells with reduced CD44 expression enhanced the malignant phenotype when compared to control cells. They suggested that the apparent discrepancy between in vitro and in vivo results highlights the critical impact of the tumor environment on OS progression [87]. A recent study has identified increased levels of total *CD44* mRNA and membrane localization of CD44 in primary and metastatic OS compared to normal bone. In addition, they showed that CD44 promotes transendothelial migration of tumor OS cells [88].

Some studies have proven that Hippo signaling dysregulation is associated with SOX2 level in OS. Basilico et al. described that SOX2 maintains cancer stem cells (CSC) in OS and antagonizes the Hippo pathway by directly repressing two Hippo activators, *NF2* and *WWC1*, leading to exaggerated YAP function. Moreover, this study showed the requirement of SOX2 for OS formation and survival of the tumor cells, proposing that disruption of these pathways initiated by SOX2 is an attractive strategy for the treatment of OS [89,90]. In addition, it has been described that YAP can regulate the expression of *SOX2* by interacting with TEAD on two TEAD-binding DNA elements near the *SOX2* gene. Thus, SOX2 and YAP reinforce each other’s expression to maintain stemness and tumorigenicity in OS [73]. The crucial role of SOX2 in OS was likewise described by Upal Basu-Roy et al., who reported that thiazolidinedione drugs (TZDs), a class of small-molecule activators of PPARγ, decrease the expression of target genes of YAP with a simultaneous reduction in SOX2 and YAP nuclear localization. They demonstrated that TZDs target the PPARγ^high^-expressing CSC population and restores the tumor-suppressive Hippo signaling effects in OS [135].

#### 3.1.3. LATS1/2

A recent study showed that the inhibition of *Tankyrase 1* (*TANK1*), classified as a positive regulator of telomere length, by antisense oligodeoxynucleotides (*TANK1-ASODN*) decreased cell growth, migration, invasion, and EMT in OS cells. Mechanistically, the inhibition of *TANK1* expression modulated the Hippo/YAP signaling, inducing significantly LATS1 expression and, subsequently, YAP phosphorylation [91]. 

Another study by Su X. et al. showed the overexpression of the *miR-100HG* in OS tissues and cell lines and the correlation with poor prognosis for OS patients. Inhibition of OS progression was observed after a *miR-100HG* knockdown by reducing cell proliferation, cell cycle distortion, and apoptosis resistance. Mechanism investigation revealed that *miR-100HG* exerted oncogenic function in OS by inactivating the Hippo signaling pathway. Concretely, RNA immunoprecipitation assay revealed the binding between *miR-100HG* and *EZH2* in OS cells, suggesting that the expression of *miR-100HG* downstream targets is inhibited by epigenetic mechanisms involving EZH2. Further experiments revealed that both *miR-100HG* and *EZH2* knockdown significantly upregulated the *LATS1*/2 expression in OS cells. Finally, ChIP assay results showed that EZH2 binding to the *LATS1/2* promoter is inhibited by *miR-100HG* silencing, and consequently, a reduction of H3K27 trimethylation is displayed [92]. 

A more recent study developed by the same research group has reported that the deubiquitinase YOD1, which stabilizes ITCH (Itchy E3 Ubiquitin Protein Ligase) and facilitates ITCH-mediated LATS1/2 ubiquitination and degradation, was highly expressed in OS cells. They described that overexpression of *miR-302b* decreased the mRNA expression of *YOD1* (direct target of *miR-302b*), *ICTH*, and *YAP1*. In contrast, LATS1 expression increased, suggesting that the YOD1-ICTH-LATS1-YAP axis is controlled by *miR-302b* [93]. 

Wu X. et al. described that the upregulation of the lysyl hydroxylase PLOD1 was correlated with the progression and worse survival probability of OS patients. Moreover, PLOD1 overexpression promoted OS tumorigenesis and metastasis in vitro and in vivo, and the mRNA levels of *CTGF* and *CYR61* were significantly upregulated. In contrast, protein levels of p-LATS1 and p-YAP were decreased without disturbing p-MST1/2. Mechanistically, they proved that *PLOD1* is directly regulated by *miR-34c* and *PLOD1* mRNA, and miR-34c levels negatively correlated in OS samples [94]. 

#### 3.1.4. RASSF

Three RASSFs (RASSF4, RASSF5, and RASSF10) proteins have been identified as tumor suppressors in OS. *RASSF5* and *RASSF10* have been reported to be epigenetically inactivated by hypermethylation of their CpG island promoters in OS. In vitro experiments in OS cell lines proved that overexpression of *RASSF4* significantly inhibited proliferation, migration, and invasion as well as the EMT process [95], and *RASSF5* overexpression markedly suppressed cell proliferation and invasion and induced cell apoptosis through activation of the MST1/LATS1 pathway [96].

#### 3.1.5. TAZ

Interestingly, although there is not much data on the potential role of TAZ on OS tumorigenesis, some studies link TAZ and miRNAs to OS oncogenic behavior. Thus, Ma J. et al. demonstrated the upregulation of TAZ in OS tissues and cell lines, and OS cell migration, invasion, and proliferation could be induced by TAZ overexpression. The mechanistic study revealed that TAZ overexpression leads to *miR-224* upregulation, which inhibits the tumor suppressor SMAD4 [136]. Similar findings were reported by Shen S. et al., which described that TAZ is upregulated in OS and modulates EMT. They demonstrated that TAZ induces *miR-135b* and suppresses the expression of *LATS2*, *APC*, and *GSK-3β* [137]. 

### 3.2. Ewing Sarcoma

Ewing sarcoma (EwS) is the second most frequent primary bone tumor and affects mainly children and young adolescents. EwS is characterized by gene fusions between *EWSR1* and members of the *ETS* gene family (usually *FLI1*), which are considered the main oncogenic driver of the disease, but exhibit a low somatic mutation rate, and secondary genetic alterations are uncommon [61,138]. No recurrent genetic alterations in members of the Hippo pathway have been described in EwS. Instead, aberrant activation of TAZ and YAP has been observed in several studies, and we have shown that it associates with poor patient prognosis [67,69,98,139]. Moreover, TAZ and YAP suppression negatively affects proliferation and invasion capacity in EwS cell lines, and YAP could also mediate resistance to contact inhibition [69,140]. 

Interestingly, we described a transcriptional antagonism between the fusion *EWSR1::FLI1* and YAP/TAZ [69], which may underlay the phenotypic plasticity of EwS cells. Franzetti G.A. et al. proposed that this plasticity relies on the expression levels of the fusion protein, with low levels favoring a migratory phenotype and, therefore, the dissemination of the disease in EwS [141]. Opposing gene expression signatures could result from interference between the fusion protein and YAP/TAZ/TEAD–AP1 complexes, as evidenced by Katschnig et al. [97], but direct or indirect transcriptional repression of TAZ by EWSR1::FLI1 could also contribute to this antagonism [69,98]. We have also speculated that Ewing sarcoma-associated transcript 1 (*EWSAT1*), a long noncoding RNA that mediates *EWSR1::FLI1* gene repression by interacting with a heterogeneous nuclear ribonucleoprotein [142], might modulate the opposing gene signatures. We observed increased *EWSAT1* mRNA expression upon YAP/TAZ silencing in the EwS cell line SK-N-MC [142].

Activation of YAP/TAZ in EwS could be mediated by epigenetic regulation of the *RASSF1* locus [69]. *RASSF1* encodes different isoforms, which affect the activity of the final Hippo effectors YAP/TAZ in opposite ways. The isoform *RASSF1A* contributes to the repression of YAP/TAZ by Hippo core kinases, whereas *RASSF1C* promotes the activation of YAP through functional interaction with SRC family kinases [143]. These two isoforms are differently regulated by the hypermethylation of the locus. Whereas *RASSF1A* is silenced, *RASSF1C* expression is induced from an alternative promoter. This may explain the correlation of DNA hypermethylation of *RASSF* genes with poor outcomes of EwS patients [99,100].

Activation of YAP by SRC has also been proposed as the mechanism mediating tenascin C (TNC) induction of Metastasis-associated lung adenocarcinoma transcript 1 (*MALAT1*), a long noncoding RNA with oncogenic properties [144]. Indeed, a feed-forward loop between TNC and SRC promotes cell metastatic behavior [145].

### 3.3. Epithelial Hemangioendothelioma

Epithelial Hemangioendothelioma (EHE) is a rare malignant vascular tumor that originates from vascular pre-endothelial or endothelial lineage cells, arising at a great variety of anatomic sites but mainly affecting lung, liver, and soft tissue with a variable clinical course [61,146]. In 2001, a chromosomal translocation t(1;3)(p36;q25) was identified in EHE [147], which was later on described as a genetic alteration generating the gene fusion *WWTR1::CAMTA1* [63,148], present in >90% of the cases and hence considered a useful genetic hallmark for differential diagnosis [149,150]. A less frequent fusion gene, *YAP1::TFE3*, is present in <10% of EHE, and those cases display a different morphology [64]. Moreover, *YAP1::TFE3* fusion seems to be associated with better patient prognosis than *WWTR1::CAMTA1* positive patients [64,102]. Additional oncogenic alterations related to DNA damage response, cell cycle, and epigenetic pathways are present in at least 20% of cases [102]. However, pathognomonic gene fusion appears as the primary oncogenic driver in EHE. 

Mechanistically, Tanas et al. have shown that WWTR1::CAMTA1 nuclear localization and TEAD-dependent transcriptional activity cannot be restrained by the Hippo pathway, and therefore the fusion oncoprotein is constitutively active [151]. Several fusion variants have been described, but all of them conserve the TEAD binding domain, 14-3-3 binding motif, and all or most of the WW domain of TAZ fused to the transactivation domain (TAD), TIG domain, ankyrin repeats, and IQ domains of CAMTA1 [101]. Besides, CAMTA1 also contributes to a non-canonical nuclear localization signal which translocates the fusion into the nucleus [151]. This results in the induction of a TAZ-like transcriptional program which promotes cellular transformation and adhesion-independent growth. Furthermore, it has been suggested that YAP/TAZ-induced transcriptome could contribute to the prominent fibrous stroma commonly observed in EHE [101].

### 3.4. Myxoid Liposarcoma

Myxoid liposarcoma (MLS), the second most common type of liposarcoma, is a malignant adipose tissue neoplasm that develops in deep soft tissues and is characterized by a chromosomal rearrangement between *FUS* and *DDIT3* genes, producing a chimeric transcription factor [152]. This genetic hallmark is considered the primary oncogenic driver of the disease [153,154]. 

A recent report identified *YAP1* in an RNA screen as an essential gene in *FUS::DDIT3*-expressing mesenchymal stem cells [103]. In addition, this study describes nuclear YAP expression in 96% of MLS human specimens and expression of the downstream targets *FOXM1* and *PLK1*. Prevalent YAP expression in MLS is further confirmed in other immunohistochemical studies [67,68]. Functional assays indicated that the oncogenic properties of FUS::DDIT3 could be mainly mediated by YAP. FUS::DDIT3 not only induces *YAP1* transcription but also promotes YAP nuclear localization and physically interacts with YAP in the nucleus, suggesting a cooperative function between both factors to modulate the transcriptional output in MLS cells [103]. It has been lately described that FUS::DDIT3 induces concurrent activation of IGF-IR/PI3K/AKT signaling and cooperates with YAP to regulate oncogenic gene sets in MLS and disrupt terminal adipogenic differentiation [104].

### 3.5. Sclerosing Epithelioid Fibrosarcoma and Low-Grade Fibromyxoid Sarcoma

Sclerosing epithelioid fibrosarcoma (SEF) is an aggressive sarcoma, classically composed of nests and cords of epithelioid cells within a dense collagenous matrix, with the presence of both large paucicellular fibrous zones and focal myxoid areas, features also seen in low-grade fibromyxoid sarcoma (LGFMS) [61,155]. LGFMS is a malignant, often late-metastasizing tumor with low to moderate cellularity and consists of bland spindle cells with small, angulated nuclei and scarce cytoplasm, typically showing an abrupt transition from myxoid to fibrous areas [61,156]. 

Conventional SEF and LGFMS are two closely related mesenchymal entities, with SEF harboring mostly *EWSR1::CREB3L1* fusions and LGFMS exhibiting *FUS::CREB3L2* fusions [157,158]. Both entities present the upregulation of MUC4, which is detectable at the protein level and used as a surrogate marker. However, a subset of cases negatives for MUC4 expression were reported to harbor complex rearrangements between *YAP1* and lysine methyltransferase 2A (*KMT2A*) loci which exhibit unifying morphologic features slightly different from conventional cases and show an aggressive behavior [105,106,107,108,109]. For these reasons, the possibility of reclassifying YAP1::KMT2A tumors with SEF-like histologic features as a distinct entity related to SEF has been raised. 

The most recent study by Massoth L.R. et al. [108] interrogated public genomic data from 14,680 sarcomas and found 33 patients with *KMT2A* rearrangements (0.2%), including 16 patients with tumors positive for *YAP1::KMT2A* fusion. Several cases were also reported to bear fusions between *KMT2A* and other partners, such as Vimentin (VIM). This study and the previous reports are coincident in reporting poor performance of FISH to detect the chromosomal aberration that could be due to the complex rearrangement with the configuration *YAP1::KMT2A::YAP1* [108]. This configuration retains the CxxC-binding domain of KMT2A, which is functionally relevant in the pathogenesis of acute leukemias [110], and the TEAD-binding domain and PDZ-binding motif of YAP.

### 3.6. Rhabdomyosarcoma

Rhabdomyosarcoma (RMS) is the most common soft tissue sarcoma (STS) in children and adolescents. The WHO [61,156] recognizes four RMS subtypes, being the two most common subtypes the embryonal and alveolar RMS (ERMS and ARMS, respectively). The presence of the *PAX3/7::FOXO1* fusion gene is detected in most ARMS cases, and it is considered the oncogenic driver of this entity. Less common fusion gene variants include the fusion of *PAX3* to *FOXO4*, *NCOA1* or *INO80D,* and *FOXO1* to *FGFR1*. In contrast to ARMS, the oncogenic drivers in ERMS are still undefined. The two rarer RMS subtypes are pleomorphic RMS (PRMS) and spindle cell/sclerosing RMS (SRMS). Gene fusions involving *VGLL2*, *SRF*, *TEAD1*, *NCOA2*, *CITED2*, *EWSR1*, *FUS*, *TFCP2,* and *MEIS* genes have been identified in some subtypes of SRMS [61,156].

#### 3.6.1. Alveolar RMS

Interestingly, *PAX3::FOXO1* gene fusion has been found to suppress the Hippo pathway in ARMS [159]. Specifically, this study revealed that RASSF4 expression was highly increased in *PAX3::FOXO1*–positive ARMS, and its expression was necessary for ARMS cell proliferation, senescence evasion, and tumorigenesis. Mechanistically, it was evidenced that the gene fusion upregulates *RASSF4*, which associates with MST1 kinase to inhibit downstream signaling in PAX3::FOXO1–positive ARMS. In addition, they showed that YAP was upregulated in both ERMS (in part to the increased copy number of *YAP1* locus) and ARMS subtypes, which suggests that Hippo pathway dysregulation is crucial for RMS tumorigenesis [65,159].

Similar studies have reported that the *RASSF1* promoter is methylated in pediatric RMS but not adult RMS [66,111]. Thus, pediatric RMS becomes a potential candidate for epigenetic modifiers that can activate *RASSF1*. Indeed, Slemmons K.K. et al. have recently proved that treatment with a DNA methyltransferase inhibitor (DNMTi) can upregulate Hippo-activators *RASSF1* and *RASSF5* by promoter demethylation in RMS. Moreover, they reported that combined treatment with DNMTi and dasatinib ablates ARMS cell growth in vitro and trends towards decreased tumor growth in vivo [112].

#### 3.6.2. Spindle Cell/Sclerosing RMS

A study of pediatric SRMS identified three different subsets with distinctive molecular features. A subset of pediatric SRMS presenting at birth or within one year of age exhibited recurrent gene fusions involving *VGLL2, SRF, TEAD1,* or *NCOA2* and appeared to be associated with a better outcome [113]. Specifically, *VGLL2* rearrangements were observed in 63% of cases of this subset (*VGLL2::CITED2* in four patients and *VGLL2::NCOA2* in two cases), and *NCOA2* rearrangements were detected in the rest of the cases (*TEAD1::NCOA2* in two cases, and *SRF::NCOA2* in one case). Subsequently, another study identified six *VGLL2::NCOA2* cases and one *VGLL2::CITED2* case also occurring in very young children [109].

The *NCOA2* gene rearrangements were reported in congenital/infantile SRMS in 2013, including a *TEAD1::NCOA2* fusion in a case located in the chest wall of a 4-week-old child [114]. Afterward, several studies reported the *TEAD1::NCOA2* gene rearrangement in a subset of pediatric SRMS, which followed a favorable clinical outcome compared to those with *MYOD1* mutations [113,115,116]. Although the *NCOA2::TEAD1* finding in pediatric SRMS has a prognostic value in clinical practice, the molecular significance of TEAD rearrangement and this involvement in the dysregulation of the hippo signaling is still unknown.

*VGLL2* belongs to the Vestigial-like (VGLL) family, whose members have been shown to interact with TEADs in overlapping binding sites for YAP and TAZ. Thus, VGLL family members function as TEAD cofactors and are involved in tumor development in various types of neoplasms [117,118,119]. *VGLL2* was identified as a *VGLL1* homolog with expression limited to the skeletal muscle lineage. VGLL2, TEAD1, and SRF are transcriptional activators of muscle-specific genes [160,161], and VGLL2-fused tumors express muscle-related genes [109]. VGLL2, TEAD1, and SRF retain most of their functional domains as 5’ partners in the gene fusions. Still, the absence of overt rhabdomyoblastic differentiation in SRMS has led to speculation that the gene fusions could block skeletal muscle differentiation to maintain a primitive phenotype [113]. Interestingly, despite *VGLL2*-fused tumors expressing some muscle differentiation markers, they are not transcriptionally related to ERMS tumors [109]. Moreover, *VGLL2::NCOA2* and *VGLL2::CITED* show some transcriptome heterogeneity, which may underlie histological differences. *VGLL2::NCOA2* tumors present low cellularity and fibrous stroma, whereas *VGLL2::CITED* tumors exhibit an SRMS-like morphology [109]. 

### 3.7. Synovial Sarcoma

Synovial sarcoma (SS) is an aggressive mesenchymal tumor that usually occurs in soft tissues. SS constitutes 8–10% of all soft tissue sarcomas, mainly affecting adolescents and young adults [162]. SS is characterized by a pathognomonic translocation between chromosomes X and 18 that involves *SS18* and *SSX* genes, commonly *SS18::SSX1* and *SS18::SSX2* [163]. 

Analysis of YAP/TAZ levels in different sarcoma cell lines and tumor samples showed that SS is one of the tumors with higher levels of nuclear YAP/TAZ proteins [68]. Similar to other sarcoma subtypes, the YAP/TAZ activity has been associated with the loss of Hippo kinases. In the previously cited study, Merrit et al. show that all SS-analyzed samples are negative for at least one of the kinases [122]. The presence of *SS18::SSX* translocation has also been described as a regulator of YAP/TAZ activity. In SS cell lines, the loss of *SS18::SSX* expression is associated with a reduction of YAP/TAZ-mediated transcriptional activity. In SS, the SS18::SSX-mediated dysregulation of YAP/TAZ has been linked to IGF-1R/PI3K/AKT activation, a pathway implicated in tumorigenesis in several types of cancer, through a decreased phosphorylation of LATS1 and MOB1. Because of the importance of the Hippo pathway in SS malignancy, SS cells and tumors show a high sensitivity to Verteportin, a suppressor of YAP/TAZ-TEAD binding [120].

### 3.8. Osteoblastoma

Osteoblastoma (OB) is an infrequent primary osseous tumor, locally aggressive and typically occurring in the medulla of long bones and the neural arch. A high proportion of cases present recurrent rearrangements in *FOS* or *FOSB* genes, but a subset of cases do not present these distinctive alterations [121,164]. Instead, they seem to be characterized by a homozygous deletion in chromosome band 22q12. Since the *NF2* gene localizes at this region, the authors speculate that it may play a role in the pathogenesis of that subgroup of tumors [121]. Loss of *NF2* expression could thus ultimately lead to YAP/TAZ activation, which is able to cooperate with the AP-1 transcriptional complex. As FOS is one of the main components of the AP-1 complex, the mechanisms underlying the pathogenesis of OB could be similar irrespective of the genetic alteration [121]. 

### 3.9. Undifferentiated Pleomorphic Sarcoma

Undifferentiated pleomorphic sarcoma (UPS), previously named malignant fibrous histiocytoma (MFH), is an aggressive adult sarcoma usually located in the extremities [165]. UPS is characterized by the presence of complex karyotypes, non-specific differentiation, and atypical anaplastic spindles and round cells [166]. Similar to other sarcoma subtypes, such as fibrosarcoma or liposarcoma, oncogenic driver mutations have not been described in this type of tumor [167]. 

Remarkably, YAP/TAZ stabilization has been described in UPS, and their expression has been correlated with decreased overall survival [67]. Mechanistically, deregulation of the Hippo pathway is associated with two different processes: the loss of Hippo kinases and the epigenetic repression of *AMOT* [123,124]. Because of the negative regulation of TAZ and YAP by the Hippo pathway, Merrit et al. hypothesize that the loss of Hippo kinases (MST1, MST2, LATS1, and LATS2) could be implicated in the activation of these proteins. In this study, 77% of UPS analyzed samples (20/26) were negative for at least one Hippo kinase. They also demonstrate that proteasomal degradation and epigenetic modifications, including deacetylated histones and hypermethylated promoters, are implicated in the negative regulation of Hippo kinases. These results suggest that proteasome or DNA methyltransferase/histone deacetylase inhibitors could be used in UPS patients with activation of YAP/TAZ [122]. 

Deregulation of the Hippo pathway in UPS promotes tumorigenesis through the modulation of the expression of different factors. Forkhead box M1 (*FOXM1*) is a YAP transcriptional target highly expressed in sarcomas. Downregulation of *FOXM1* in in vitro and in vivo sarcoma models reduces cell proliferation and sarcomagenesis [168]. In UPS, FOXM1 expression has been associated with the development of metastases in mouse models [169]. FOXM1 also induces the expression of pluripotency-related genes. Similar to embryonic carcinoma or neuroblastoma, FOXM1 in UPS could maintain the characteristic undifferentiated state of this sarcoma [170]. Different strategies have been developed targeting FOXM1 that could be used for the treatment of UPS patients. Thiostrepton, a proteasome inhibitor, efficiently reduces the expression of FOXM1, suppressing tumor growth in fibrosarcoma models [168].

High levels of YAP in UPS tumors have also been associated with the upregulation of the NF-kB factor. NF-kB is expressed in normal myoblast, the most accepted cell-of-origin of UPS, promoting proliferation and an undifferentiated state [171,172]. Shuai Ye et al. described that YAP-related regulation of NF-kB depends on Ubiquitin Specific Peptidase 31 (USP31), a negative regulator of NF-kB expression repressed by YAP. In this study, repression of USP31 induced more NF-kB activity, promoting proliferation and reducing the differentiation capacity. They also show that the use of epigenetic modulators such as Vorinostat/SAHA and JQ1 reduces the expression of YAP and, in consequence, the pathogenic effects of the protein in UPS models [124]. Finally, the same authors discovered that YAP is implicated in UPS tumorigenesis blocking autophagy in NF-kB independent manner and repressing circadian clock activity through NF-kB upregulation. Circadian clock genes promote the expression of unfolded protein response (*UPR*) genes. Loss of UPR activity in UPS could be associated with the undifferentiated state of this tumor [173]. 

The interaction between the UPS cells and extracellular matrix components, such as hyaluronic acid (HA), has also been associated with tumorigenesis and metastatic capacity. The expression of the hyaluronan-mediated mobility receptor (*HMMR*) gene, which encodes HA surface receptor RHAMM, is activated by YAP and TGFβ signaling (upregulated in UPS). In addition, it has been reported that the loss of YAP/TGFβ activity in UPS animal models reduces the invasion and migration of tumor cells [123].

### 3.10. Chondrosarcoma

Chondrosarcomas (CS) are groups of locally aggressive or malignant neoplasms that produce a cartilaginous matrix and represent the second most common primary bone tumor [174]. A recent report describes the elevated expression of protein arginine methyltransferase 1 (PRMT1) and nuclear accumulation of YAP in CS specimens. Furthermore, PMRT1 and YAP were positively correlated and associated with high histologic grade and shorter overall survival, being YAP an independent prognostic marker of poor survival [125]. Accordingly, a previous report had also described higher frequencies of YAP and TAZ IHC expression in high-grade CS specimens [67]. PRMT1 is the predominant type I PRMT in mammalian cells, accounting for at least 85% of all arginine methylation in human cells, with implications in several types of cancer [175]. Functional assays in the study by Chen et al. revealed that PMRT1 promoted CS cell growth through suppression of apoptosis, and this could be mediated in part by activation of YAP. PMRT1-dependent activation of YAP was reported to involve LATS1 [125].

### 3.11. Ossifying Fibromyxoid Tumor

Ossifying fibromyxoid tumor (OFMT) is a rare soft tissue neoplasm of an uncertain line of differentiation and intermediate risk of malignancy. Up to 85% of OFMT present recurrent rearrangements mostly involving PHD finger protein 1 (PHF1), a Polycomb group protein, but also translocations of other genes related to histone modification functions as well [176,177]. A transcriptome sequencing study assessed the presence of alternate gene fusions in a subset of cases lacking those translocations [126]. Two novel gene fusions were identified, *CREBBP::BCORL1* and *KDM2A::WWTR1*. KDM2A is a histone demethylase with a prominent role in the cell proliferation of mesenchymal stem cells. Interestingly, transcriptional profiling grouped OFMT cases with different gene fusions, except the case with *KDM2A::WWTR1*, which clustered with other tumor types [126].

## 4. Targeting the Hippo Pathway as a Therapeutic Approach for Sarcomas

The potential of the Hippo Signaling Pathway activation/inhibition as a prognostic indicator and its key role in CSC renewal, tumor growth, migration, and invasion in several types of cancers, including sarcomas, has led many research groups to develop diverse strategies targeting YAP/TAZ network for anti-cancer therapy. Furthermore, it has been described that YAP/TAZ upregulation is involved in mechanisms inducing drug resistance, and YAP levels might limit the clinical efficacy of RAF and MEK inhibitors in melanoma [178]. Likewise, Li et al. described the link between the Hippo pathway and CDK4/6 inhibitors resistance in breast cancer cells. Mechanistically, they revealed that the Hippo pathway is suppressed because of *FAT1* loss, and subsequently, YAP and TAZ bind to the CDK6 promoter and upregulate its expression, promoting drug sensitivity [179]. In the same way, it has been suggested the potential combination strategy of CDK4/6 and IGF1R inhibitors for EwS, due to IGF-1R signaling activation, has been reported as a CDK4/6 drug resistance mechanism [180]. 

Interestingly, some small molecule inhibitors or drugs have been discovered to modulate Hippo pathway activity directly or indirectly at various levels. In this review, we will focus on those molecules that target the Hippo Signaling Pathway and are being tested in cancer clinical trials, particularly in sarcomas (Table 2).

### 4.1. Inhibition of YAP-TEAD Interaction: Verteporfin

The most used molecule is verteporfin, a benzoporphyrin-derived compound that has been approved by the FDA for the photodynamic treatment of age-related neovascular macular degeneration [181]. Verteporfin is the only reported direct inhibitor of YAP/TAZ. It was described that verteporfin binds to YAP and changes its conformation, inhibiting the binding of YAP-TEAD [182]. Later, it was reported that verteporfin increases 14-3-3σ levels, which promotes the translocation of YAP from nuclear to cytoplasm, decreasing its transcriptional co-activation function [183]. A recent study has revealed a mechanism by which the function of YAP is inhibited by verteporfin by regulating YAP SUMOylation in endometrial cancer. They also described that Serine127 phosphorylation of YAP is important for YAP sumo modification [184]. At the transcriptional level, verteporfin has been described to reduce the expression of Hippo pathway targets genes, and in vitro and in vivo studies have proven that verteporfin decrease proliferation and migration, and invasion of certain cancer cells [182,185,186,187,188,189,190], including EwS and SS cells [98,120]. Furthermore, Visudyne, the FDA-approved liposomal formulation of verteporfin, is being tested in some clinical trials, such as the treatment of cutaneous metastases of breast cancer [191].

### 4.2. YAP/TAZ Cytoplasmic Retention: Dasatinib, Statins, Pazopanib, and Metformin

A small molecule screening carried out by Oku et al. in 2015 showed that dasatinib, statins, and pazopanib inhibited the nuclear localization of YAP/TAZ and TEAD-dependent transcription, and induced YAP/TAZ phosphorylation in breast cancer cell lines [192]. 

Dasatinib was originally described as an SRC kinase inhibitor and then shown to inhibit Bcr-Abl and other tyrosine kinases. It has been reported that dasatinib blocks cell migration and invasion in many diverse human sarcoma cell lines and induces apoptosis in the bone sarcoma subgroup through inhibition of SRC-mediated signaling [193]. Numerous studies have reported that YAP and TAZ can be activated and stabilized by SRC-family kinases -mediated phosphorylation [60]. Dasatinib has shown antitumor efficacy in several types of sarcomas, including alveolar soft part sarcoma (ASPS) [194], uterine leiomyosarcoma (LMS) [195], neuroblastoma, EwS [69,196], childhood RMS [112] and uterine sarcoma [197]. Indeed, dasatinib is being tested in several clinical trials in cancer, highlighting chronic myeloid leukemia [198,199,200], acute lymphoblastic leukemia in adults [201], metastatic breast carcinoma [202], lung cancer [203,204], and several types of sarcomas [205,206,207,208,209].

Statins are reductase-competitive inhibitors that are commonly used to treat hypercholesterolemia by inhibiting the mevalonate pathway. They function by suppressing hydroxymethylglutaryl-coenzyme A (HMG-CoA) reductases, the rate-limiting enzymes in the synthesis of a fatty acid intermediate named mevalonate [210]. Aberrant inactivation of the mevalonate pathway has been reported to promote tumor progression and has a marked negative effect on YAP/TAZ transcriptional activity, as YAP/TAZ actions need mevalonate, geranylgeranyl pyrophosphate (GGPP) and Rho GTPases [30]. Many studies have demonstrated that statin use could exhibit potential survival benefits for cancer patients and appeared to be very promising in combined therapies, as they have been shown to reduce the resistance of cancer cells to other anti-cancer drugs [210,211,212,213,214]. Statin antitumoral effects have also been demonstrated in fibrosarcoma and OS cell lines [215,216]. Accordingly, a protective role in breast-cancer-related mortality [214], an improvement in ovarian cancer survival and multiple myeloma [217,218], and a reduction of the risk of developing lethal prostate cancer [219] have been observed among statin users. In this same context, a strong association between preoperative statin therapy and reduced postoperative mortality following surgical resection for rectal cancer has been reported [220]. Furthermore, statin treatment in chronic obstructive pulmonary disease (COPD) may reduce the risk of lung cancer [221]. Consequently, statins are being tested in several cancer clinical trials, such as oesophageal adenocarcinoma [222] and rectal cancer [223].

Pazopanib is a c-KIT, FGFR, PDGFR, and VEGFR multi-kinase inhibitor, but it has also been proved that it induces proteasomal degradation of YAP and TAZ [192,224,225,226]. Pazopanib has shown potent antitumor activity in many cancer cells [224,227,228,229] and is being tested in a myriad of clinical trials as an anti-cancer therapy for lung [230], ovarian [231,232,233], prostate [234], renal cell carcinoma [235], urothelial [236], and several types of sarcomas [237,238,239,240,241,242,243,244,245,246,247,248].

It is well-known that Metformin (MET) stimulates AMP-activated protein kinase (AMPK) and is widely used for the treatment of hyperglycemia. However, recent studies have described that MET interferes with the Hippo signaling pathway. Wu et al. have reported that MET activates the AMPKα, which alters the YAP/TEAD4/CCNE1/2 axis signaling, inducing cell cycle arrest and reducing cell growth of bladder cancer cells [249]. Jin et al. showed that MET controls miR-381/YAP activity and reduces the malignant phenotype of non-small cell lung cancers (NSCLCs) cells [250]. Another mechanism has been reported by Liu et al. where MET induces activation of the Hippo pathway through Scribble (SCRIB). Upregulation of SCRIB expression recruits MST1/2 and LATS1 to the plasma membrane, leading to YAP phosphorylation and its retention within the cytoplasm and finally inhibiting cell proliferation and invasion in human breast cancer cell lines [251]. Another recent study described that MET treatment downregulated YAP/TAZ expression and enhanced YAP phosphorylation in melanoma cells [252]. Thus, recent studies have examined the potential use of MET in cancer patients to decrease tumor growth, reduce the risk of cancer and improve prognosis [253,254,255]. The anti-cancer effects of MET treatment have also been observed in several types of sarcoma cell lines, such as OS [256,257,258,259,260], EwS [259,261], RMS [259,262], and endometrial [263]. In addition, MET is currently under several clinical trials in cancer, including colorectal [264], endometrial [265], ovarian [266], esophageal [267], and CS [268].

### 4.3. Inhibition of TEAD-Transcription Activity

TEAD transcription factors (TEAD1-4), as the downstream effectors for YAP/TAZ activity, are very attractive therapeutic targets to disturb Hippo-induced transcriptional activity. They are composed of two well-structured and conserved domains, the YAP-binding domain (YBD) and the DNA binding domain (DBD), separated by a proline-rich region [269]. The YBD is stabilized by S-palmitoylation and is required for its function in hippo pathway signaling [270]. Thus, TEAD lipidation status is a regulator of protein homeostasis, and its modulation can be regulated by small molecules [271,272].

Remarkably, a small molecule inhibitor of TEAD, IK-930, that prevents palmitate binding has been very recently described. In preclinical models, IK-930 demonstrates antitumor activity in mouse xenograft models with Hippo pathway genetic alterations such as *NF2* deficiency and gene fusion involving *YAP1* and *WWTR1*. IK-930 is under clinical investigation, Phase 1, as an oral TEAD inhibitor agent in patients with advanced solid tumors. This study began in January 2022 and is currently recruiting [273].

## 5. Conclusions

The Hippo pathway signaling represents a potential opportunity for cancer treatment. As has been discussed in this review, the Hippo pathway is dysregulated in many types of sarcomas and has been associated with tumor progression, malignancy, and poor prognosis. The research efforts for unveiling the Hippo pathway implications in sarcoma development and clinical behavior will provide new therapeutic insights. The identification of new drugs targeting this signaling pathway is, to date, a challenge for pharmaceutical companies and the sarcoma community.

## Figures and Tables

**Table 1:** Deregulation mechanisms of Hippo pathway in sarcomas.

Sarcoma Subtype	T-Sarcoma/ Non T-Sarcoma	Hippo Member	Deregulation Mechanism	Deregulating Factors or Genetic Aberration	References
OS	Non T-Sarcoma	YAP	Transcriptional	H19 lncRNA/ Hedgehog signalling	[72]
				SOX2	[73]
			Post-transcriptional	B4GALT1-AS1 lncRNA/HuR	[74,75]
			Epigenetic	circFAT1/miR-375	[76]
				miR-625	[77]
				Gankyrin/ miR-200a	[78]
			Post-translational	FAT10	[79]
				ROCK2	[80,81,82]
		NF2	Mutation	NF2	[83,84]
			Post-translational	CD44	[85,86,87,88]
			Transcriptional	SOX2	[89,90]
		LATS1/2	Protein Upregulation	Tankyrase 1	[91]
			Epigenetic	miR-100HG/EZH2	[92]
			Post-translational	miR-302b/YOD1	[93]
				miR-34c/PLOD1	[94]
		RASSF 4/5/10	Epigenetic	Promoter hypermethylation	[95,96]
EwS		YAP	Transcriptional Interference	*EWSR1::FLI1*	[69,97]
	T-Sarcoma	TAZ	Transcriptional Repression	*EWSR1::FLI1*	[69,98]
		RASSF1/2	Epigenetic	Promoter hypermethylation	[69,99,100]
EHE	T-Sarcoma	TAZ	Chromosomal Rearrangement	*WWTR1::CAMTA1*	[63,99,100,101]
		YAP	Chromosomal Rearrangement	*YAP1::TFE3*	[64,102]
MLS	T-Sarcoma	YAP	Transcriptional induction and nuclear localization	*FUS::DDIT3*	[103,104]
SEF and LGMFS (MUC4-)	T-Sarcoma	YAP	Chromosomal Rearrangement	*YAP1::KMT2A*	[105,106,107,108,109,110]
ARMS	T-Sarcoma	MST1	Protein inhibition by indirect fusion-dependent Mechanism	*PAX3::FOXO1*-dependent upregulation of RASSF4	[14]
		RASSF1/5	Epigenetic	Promoter hypermethylation	[66,111,112]
SRMS	T-Sarcoma	TEAD	Chromosomal Rearrangement	*TEAD1::NCOA2*	[113,114,115,116]
		VGLL2	Chromosomal Rearrangement	*VGLL2::NCOA2, VGLL2::CITED*	[113,117,118,119]
SS	T-Sarcoma	MST1, MOB1	Protein inhibition by indirect fusion-dependent mechanism	*SS18::SSX*-dependent IGF-II/IGF-IR signaling loop	[120]
non-FOS-rearranged OB	Non T-Sarcoma	NF2	CNA	NF2 homozygous deletion	[121]
UPS	Non T-Sarcoma	MST1/2 and LATS1/2	Post-translational and epigenetic	Proteasomal degradation, deacetylated histones and hypermethylated promoters	[122,123,124]
		AMOT	Epigenetic	Histone deacetylation	[124]
CS	Non T-Sarcoma	LATS1 and other kinases	Post-translational	PMRT1	[125]
OFMT	T-Sarcoma	TAZ	Chromosomal Rearrangement	*KDM2A::WWTR1*	[126]

ARMS: Alveolar Rhabdomyosarcoma; CS: Chondrosarcoma; EHE: Epithelioid Hemangioendothelioma; EwS: Ewing sarcoma; LGMFS: and Low-grade Fibromyxoid Sarcoma; MLS: Myxoid liposarcoma; OB: Osteoblastoma; OFMT: Ossifying fibromyxoid tumor; OS: Osteosarcoma; RMS: Rhabdomyosarcoma; SEF: Sclerosing Epithelioid Fibrosarcoma; SS: Synovial Sarcoma and UPS: Undifferentiated Pleomorphic Sarcoma. T-sarcoma: translocation-associated sarcomas; CNA: copy number alteration.

**Table 2:** List of Hippo pathway-regulators under clinical investigation for treating sarcomas. Source: ClinicalTrials.gov (accessed on 14 November 2022).

Small Molecule	Sarcoma	Phase	ClinicalTrials.gov Identifier	Status
Dasanitib	GIST Stage III/IV Soft Tissue Sarcoma	I	NCT01643278	Completed
	RMS, Malignant PNST, CS, EwS, ASPS, Chordoma, Epithelioid Sarcoma, GSCB, HPC, GIST	II	NCT00464620	Completed with results
	Sarcoma and other tumors	II	NCT00788125	Completed with results
	RMS, ARMS, ERMS	I/II	NCT03041701	Completed with results
	GIST	II	NCT00568750	Completed
Statins (Simvastatin)	CCS, EwS, OS, RMS and other tumors	I	NCT02390843	Completed
Pazopanib	Advanced Soft Tissue Sarcoma	I/II	NCT01975519	Completed with results
	Soft Tissue Sarcoma	II	NCT02300545	Completed with results
	Sarcoma	II	NCT01593748	Completed with results
	Soft Tissue Sarcoma	III	NCT00753688	Completed with results
	Soft Tissue Sarcoma	II	NCT00297258	Completed with results
	Stage IIA/III/IV Adult Soft Tissue Sarcoma	NA	NCT01446809	Completed with results
	Adult/Recurrent LPS Recurrent/Metastatic OS Recurrent/Stage IV Adult Soft Tissue Sarcoma	II	NCT02357810	Completed with results
	Adult ASPS, Angiosarcoma, DSRCT, EHE, Epithelioid Sarcoma, EMSC, Extraskeletal OS, Adult FS, LMS, LPS, Malignant PNST, RMS, SS, UPS, Malignant HPC, Recurrent/Stage III/ IV Adult Soft Tissue Sarcoma	II	NCT01532687	Completed with results
	Adult Angiosarcoma, Recurrent / Stage III/IV Adult Soft Tissue Sarcoma	II	NCT01462630	Completed with results
	Recurrent Uterine Corpus Sarcoma and other tumors	II	NCT01247571	Completed with results
	Advanced Angiosarcoma	III	NCT02979899	Completed with results
	Surgically and metastatic LPS	II	NCT01506596	Completed with results
	Advanced/ Metastatic LPS	II	NCT01692496	Completed with results
	CS, Metastatic CS	II	NCT01330966	Completed with results
	Solid Tumors	II	NCT01956669	Completed with results
	Solid Tumor	I	NCT01468922	Completed with results
Metformin	OS, EwS	II	NCT04758000	Recruiting
	CS and other tumors	I/II	NCT02496741	Completed
	Angiosarcoma and other tumors	II	NCT01042379	Recruiting
IK-930	Adult Solid Tumor, EHE, Solid Tumors With *YAP1/TAZ* Fusion Genes, NF2 Deficiency or *YAP1* or *TAZ* Gene Fusions, and other tumors	I	NCT05228015	Recruiting

ASPS: Alveolar Soft Part Sarcoma; CCS: Clear Cell Sarcoma; DSRCT: Desmoplastic Small Round Cell Tumor; EMCS: Extraskeletal Myxoid Chondrosarcoma; ERMS: Embryonal Rhabdomyosarcoma; FS: Fibrosarcoma; GIST: Gastrointestinal Stromal Tumor; GSCB: Giant Cell Tumor of Bone; HPC: Hemangiopericytoma; LMS: Leiomyosarcoma; LPS: Liposarcoma; PNST: Malignant Peripheral Nerve Sheath Tumor. Not Applicable (NA). All clinical trials, except withdrawn, with dasatinib, statins, metformin in sarcoma patients are listed. Only completed with results clinical trials using pazopanib are shown. Phase is used to describe trials without FDA-defined phases, including trials of devices or behavioral interventions as described in https://clinicaltrials.gov/.
